# Deletion of concurrent chemotherapy on the basis of sequential chemoradiotherapy for non‐metastatic stage T4 nasopharyngeal carcinoma in IMRT era

**DOI:** 10.1002/cam4.6578

**Published:** 2024-03-08

**Authors:** Yuming Zheng, Fen Xue, Dan Ou, Xiaoshuang Niu, Chaosu Hu, Xiayun He

**Affiliations:** ^1^ Department of Radiation Oncology Fudan University Shanghai Cancer Center Shanghai China; ^2^ Department of Oncology Shanghai Medical College Shanghai China; ^3^ Shanghai Clinical Research Center for Radiation Oncology Shanghai China; ^4^ Shanghai Key Laboratory of Radiation Oncology Shanghai China

**Keywords:** deleting of concurrent chemotherapy, intensity‐modulated radiotherapy, nasopharyngeal carcinoma, T4

## Abstract

**Purpose:**

Intensity‐modulated radiotherapy (IMRT) combined with concurrent chemotherapy is deemed as the mainstay treatment in locoregionally advanced nasopharyngeal carcinoma (NPC). Nevertheless, the tolerance of severe acute toxicity of concurrent chemotherapy was unsatisfied. In addition, T4 is the predicting factor of poor prognosis for NPC patients. In this retrospective analysis, the long‐term outcomes IMRT combined by induction chemotherapy deleting concurrent chemotherapy with or without adjuvant chemotherapy for T4 non‐metastatic NPC were analyzed.

**Materials and Methods:**

From January 2005 to November 2016, a total of 145 biopsy‐proven non‐metastatic T4 NPC was treated with IMRT combined by induction chemotherapy with or without adjuvant chemotherapy. The survival and side effects of the patients were analyzed.

**Results:**

Median follow‐up time was 74 months (ranges, 8–186 months). 10.0%, 61.3%, 27.3%, and 1.3% developed grade 1, 2, 3, and 4 mucositis during IMRT, respectively. 5.5% and 2.0% patients experienced grade 1 and 2 nausea and vomiting; no patients developed grade 3 or 4 nausea and vomiting. Of 145 patients enrolled, 5‐year and 10‐year overall survival(OS) rates were 73.7% and 53.9%, local progression‐free survival(LPFS) rates were 86.1% and 71.6%, regional progression‐free survival(RPFS) rates were 96.7% and 92.8%, distant metastasis‐free survival (DMFS) rates were 86.7%, 78.2%, respectively. At the last follow‐up, five patients developed cranial nerve injury, one patient developed mandibular bone necrosis, four patients developed temporal lobe injury, four patients developed nasopharyngeal massive hemorrhage (three cases after recurrence and one case without recurrence), and five patients developed second primary tumor.

**Conclusion:**

The survival outcomes of treating T4 NPC IMRT combined by induction chemotherapy deleting concurrent chemotherapy with or without adjuvant chemotherapy are encouraging. Moreover, mucosal reaction, nausea, and vomiting reaction were reduced during IMRT.

## 1 INTRODUCTION

Nasopharyngeal carcinoma (NPC) is prevalent in South China and Southeast Asia. According to the latest statistics of GLOBAL CANCER OBSERVATORY,^1^ there were 62,444 new diagnosed cases of NPC in China, which exceeding the sum of all other Asian countries. Approximately 13.7%–26.5% of NPC were classified as stage T4 when initially diagnosed.^2^, ^3^, ^4^, ^5^ Radiotherapy is recognized worldwidely as the primary treatment for non‐metastatic NPC and T4 disease is closely associated with poor prognosis. Based on previous literature reports, the 5‐year survival rate of T4 NPC was 56.5%–68.2%, which is significantly inferior to T1, T2, and T3 NPC.^3^, ^4^, ^5^, ^6^, ^7^

Concurrent chemoradiotherapy was recommended for locally advanced NPC based on the results of two‐dimensional conventional radiotherapy (2D‐CRT) era.^8^ While in the era of intensity‐modulated radiation therapy (IMRT), improved treatment outcomes and lessened side effects were obtained for NPC patients due to the advantages of distributing highly conformal dose to tumor while sparing the adjacent organs at risk (OARs). Concurrent chemotherapy has not been demonstrated to further improve the efficacy (especially the local control for NPC) from data of previously retrospective studies using IMRT, but with increased acute and chronic reactions such as mucositis and xerostomia.^2^, ^9^, ^10^, ^11^ Multiple studies have revealed that induction chemotherapy (IC) and adjuvant chemotherapy (AC) can improve efficacy.^12^, ^13^, ^14^, ^15^ When T4 NPC patients suffer from local recurrence after primary treatment, it incurs risks of heavier side effects and graver complications receiving re‐treatment.^16^ Therefore, we aimed at investigating the long‐term efficacy and toxicities of IC followed by IMRT alone with or without AC in stage T4 NPC.

## 2 MATERIALS AND METHODS

### 2.1 Patients

From January 2005 to November 2016, 145 biopsy‐proven non‐metastatic T4 NPC patients at Fudan University Shanghai Cancer center were retrospectively enrolled. All patients received sequential chemoradiotherapy (IC + IMRT ± AC). Written consents were obtained from all patients, and the study was approved by our institutional Ethics Review Board. Initial workup included complete medical history, physical examination, biochemical and hematological blood tests, nasopharyngeal endoscopy, nasopharyngeal contrast‐enhanced magnetic resonance imaging (MRI), neck contrast‐enhanced computed tomography (CT)/MRI, and positron emission tomography‐CT (PET‐CT) or a combination of chest CT, abdominal ultrasound/CT and bone scintigraphy. Additional investigations were only performed for those with suspicious findings. Dental extraction, if deemed necessary, was performed before radiation therapy. All patients enrolled were re‐staged according to eighth edition of AJCC staging system.

### 2.2 Treatment

#### 2.2.1 Radiotherapy

A thermoplastic mask was used to secure the head, neck, and shoulders of the patients in supine position. Enhanced CT scan was performed to acquire slices from the cranial apex to the 2 cm below lower margin of the clavicle head. The Gross tumor volume (GTV) included nasopharyngeal primary tumor and metastasis lymph nodes. Clinical target volume (CTV) includes GTV and subclinical lesions. All the GTV and CTV must be expanded in all directions by 5 mm as planning target volume (PTV) and then appropriately modified accounting for the patient motion and set‐up error. The prescription dose of primary tumor was 66–70.4 Gy in 30–32 fractions; the prescribed dose was 66 Gy for positive cervical lymph nodes, 60 Gy for high‐risk area, and 54 Gy for low‐risk area in the same fraction of the primary tumor.

#### 2.2.2 Chemotherapy

All patients received IC followed by IMRT alone with or without AC. There are four species of platinum‐based chemotherapy regimens, including fluorouracil with cisplatin (PF: DDP 25 mg/m^2^ d1‐3 + 5‐FU 500 mg/m^2^/d d1 with 120‐h infusion), gemcitabine with cisplatin (GP: gemcitabine 1.0 g/m^2^ d1, d8 + DDP 25 mg/m^2^ d1‐3), docetaxel with cisplatin (TP: docetaxel 60 mg/m^2^ d1 + DDP 25 mg/m^2^ d1‐3), and docetaxel with cisplatin plus fluorouracil (TPF: docetaxel 60 mg/m^2^ d1 + DDP 25 mg/m^2^ d1‐3 + 5‐FU 500 mg/m^2^/d with 120‐h infusion). IMRT was initiated 3 weeks after completion of the IC, as well as AC was administered 4 weeks after radiotherapy. Before each cycle of chemotherapy and during the intermission of chemotherapy, patients performed complete blood count, and blood biochemical parameters. If grade 4 myelosuppression occurs, 20% dose reduction should be administered in the next cycle of chemotherapy. Chemotherapy would be delayed if the hematologic parameters of patients were not reach to standard. Up to a 2‐week delay of chemotherapy is allowed, or the chemotherapy will be terminated.

### 2.3 Evaluation and follow‐up

Follow‐up intervals: every 3 months within 2 years after completion of treatment, every 6 months from third year through fifth year and annually thereafter. Contents of each follow‐up included physical examination, nasopharyngeal MRI, neck CT/MRI, nasopharyngeal endoscopy, and plasma EBV DNA (2018 till now). Chest CT and abdominal ultrasound/CT should be performed at least every 6 months or annually according to patient status. If necessary, increase the inspection frequency and conduct PET‐CT inspection.

#### Statistics

SPSS 26.0 (SPSS Inc) software was used for data processing. Kaplan–Meier method was used to calculate local progression‐free survival (LPFS), regional progression‐free survival (RPFS), distant metastasis‐free survival (DMFS) and overall survival (OS).

## 3 RESULTS

### 3.1 Patient characteristics and treatment compliance

There were 145 patients in the whole group, of which 117 were males and 28 were females. The median age was 50 years (range, 21–72 years). The course of disease before treatment in 88 cases was within 6 months, including 53 cases within 3 months. All patients received IC in 2 or 3 cycles with TP, PF, GP, or TPF regimen. As for IMRT course, all completed at the prescribed dose except one did not complete the plan due to non‐medical reasons (63.8 Gy/29 F). After IMRT, AC were administrated to 79.3% (115) patients. Details are summarized in Table 1.

**TABLE 1:** Patient characteristics of T4 nasopharyngeal carcinoma.

Characteristics	Number (%)
Age (years)	50 (21–72)
Sex	
Male	117 (80.7)
Female	28 (19.3)
Karnofsky performance status score	
70	16 (11.0)
80	71 (49.0)
90	58 (40.0)
Course	
≥6 months	57 (39.3)
≥3 months, <6 months	35 (24.1)
<3 months	53 (36.6)
N category	
N0	20 (13.8)
N1	55 (37.9)
N2	51 (35.2)
N3	19 (13.1)
Chemotherapy regimen	
TP	6 (4.1)
PF	25 (17.2)
GP	28 (19.3)
TPF	83 (57.2)
Others	3 (2.2)
IC cycles	
2 cycles	138 (95.2)
3 cycles	7 (4.8)
AC cycles	
0cycle	30 (20.7)
1cycle	36 (24.8)
2cycles	79 (54.5)
Radiation dose (Gy)	
<66	1 (0.7)
≥66, <70.4	11 (7.6)
70.4	133 (91.7)

### 3.2 Outcomes

The median follow‐up time was 74 months (range, 8–186 months). Among 57 patients of treatment failures, 31 cases were local recurrence, 8 cases were regional recurrence, and 30 cases were distant metastasis (Table 2).

**TABLE 2:** Patterns of treatment failure.

Pattern	No.	%
Local recurrence alone	22	38.6
Reginal recurrence alone	1	1.8
Distant metastasis	23	40.4
Local recurrence + Reginal recurrence	4	7.0
Local recurrence + Distant metastasis	4	7.0
Reginal recurrence + Distant metastasis	2	3.5
Local recurrence + Reginal recurrence + Distant metastasis	1	1.8

**FIGURE 1:**
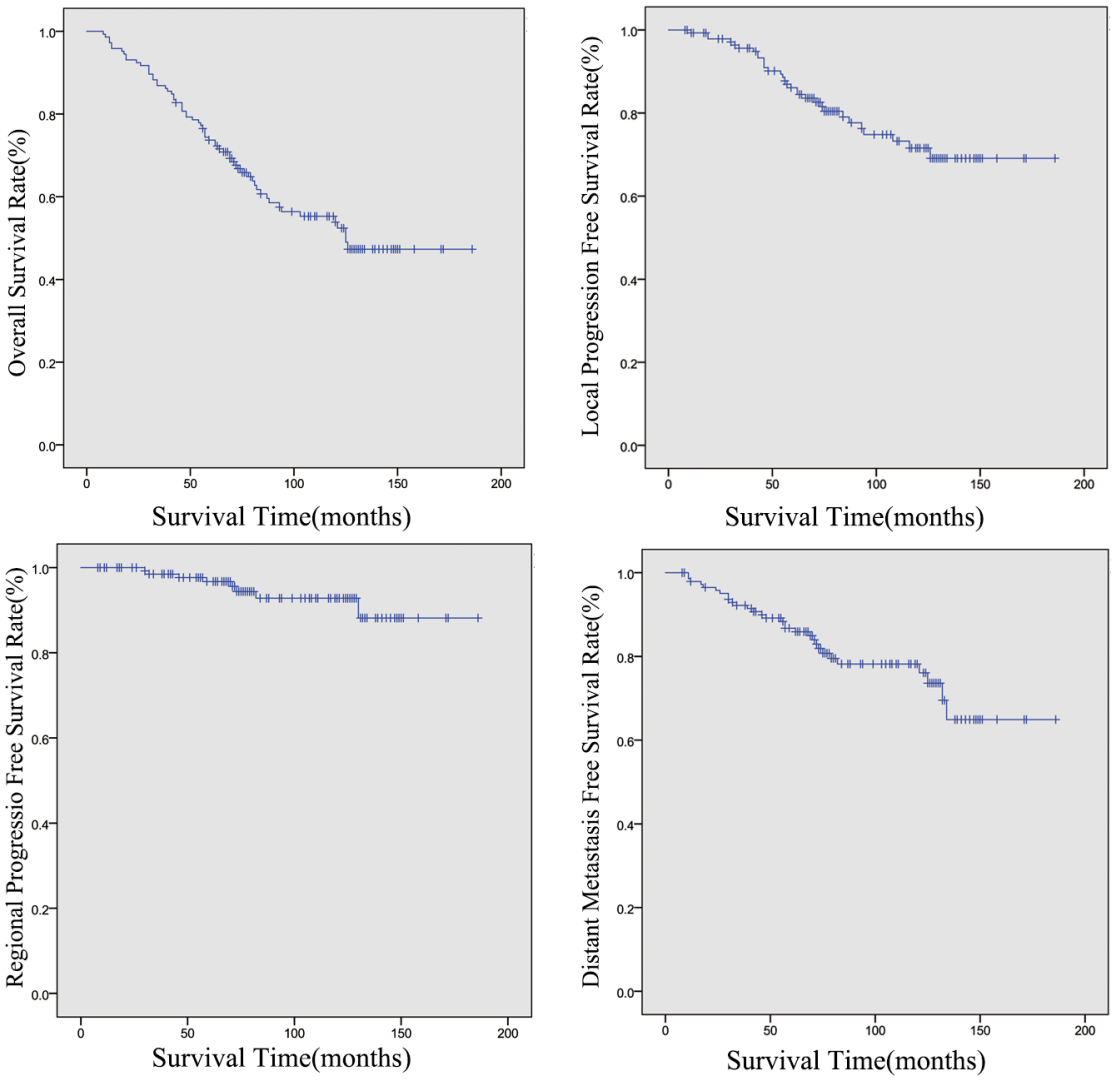
Kaplan–Meier curves showing overall survival (OS) rates, local progression‐free survival (LPFS) rates, regional progression‐free survival (RPFS) and distant metastasis‐free survival (DMFS) for 145 patients with T4 nasopharyngeal carcinoma.

Sixty‐three patients died at last follow‐up, 18 patients died of local recurrence, 18 patients died of distant metastasis, 4 patients died of local and reginal recurrence, 7 patients died of recurrence and distant metastasis, 3 patients died of second primary tumor, 3 patients died of severe nasopharyngeal bleeding, the left patients died of others reasons. 5‐year and 10‐year OS rates were 73.7% and 53.9%, LPFS rates were 86.1% and 71.6%, RPFS rates were 96.7% and 92.8%, DMFS rates were 86.7% and 78.2%, respectively (Figure 1). N stage was significantly correlated with OS and DMFS. The N3 mortality rate (14/19 cases) was high, and the estimated 5‐year and 10‐year survival rates were 52.6% and 20.8%, respectively. For all enrolled patients, 30 suffered metastasis, and the metastasis rate of N3 was high (11/19). The rates of DMFS at 5 years and 10 years were 61.3% and 37.4%, respectively. The prognosis of T4N3 was significantly worse than T4N0–2 (Table 3).

**TABLE 3:** Treatment outcomes by N stage.

	OS (%)			*p* value	DMFS (%)			*p* value
N stage	No.	5‐year	10‐year	0.008	No.	5‐year	10‐year	0.000
N0	7/20	75.0	59.1		1/20	93.8	93.8	
N1	24/55	78.0	54.1		11/55	88.5	80.9	
N2	18/51	76.4	65.6		7/51	91.3	85.8	
N3	14/19	52.6	20.8		11/19	61.3	37.4	

### 3.3 Treatment complications

There were no treatment‐related deaths during the follow‐up. According to RTOG grading criteria, the incidences of grade 1, 2, 3, and 4 acute mucositis were 10.0%, 61.3%, 27.3%, and 1.3%, respectively. 52.0% patients received fluid therapy, median fluid therapy time was 6 days (range, 1–18 days), and median weight loss was 8% (range, 0%–30.8%). None of the patients placed a stomach tube. The main late reactions were grade 1–2 xerostomia and hearing impairment; in addition, there are five patients developed cranial nerve injury, one patient developed mandibular bone necrosis, four patients developed temporal lobe injury, four patients developed nasopharyngeal massive hemorrhage (three cases after recurrence and one case without recurrence), and five patients developed second primary tumor (leukemia, liver cancer, lung adenocarcinoma, hypopharyngeal cancer, and esophageal cancer).

## 4 DISCUSSION

Radiation experts are in the progress of exploring new treatment scheme for providing equivalent survival benefits with reduced adverse effects and financial burden. The European Society for Medical Oncology (ESMO) Precision Medicine Working Group proposed several schemes of de‐intensifications: ① deletions: forgo one segment of the standard treatment; ② shortened duration of treatment: lessen treatment‐related burden and risk of toxicities by reducing treatment duration; ③ combination of the two methods above.^17^ Stage III colon cancer, breast cancer, and oropharyngeal cancer have made obvious achievements in this respect.^18^, ^19^, ^20^ André et al. conducted a prospective pooled analysis of six randomized phase 3 trials. With a median follow‐up of 72.3 months, 3‐month of adjuvant chemotherapy was not significantly inferior to 6‐month in stage III colon cancer patients in terms of overall survival, and the 5‐year overall survival results in favor of 3‐month adjuvant chemotherapy should be considered for adoption in clinical context. This conclusion demonstrates that shortened treatment duration is associated with significant reductions in associated toxicity, inconvenience, and cost.^18^ FAST Forward trial enrolled 4096 breast cancer patients (pT1‐3, pN0‐1, M0) at 97 hospitals in the United Kingdom. In these patients who were at least 18‐year‐old and underwent breast conservation surgery or mastectomy, median follow‐up was 71.5 months. The 5‐year local tumor control of this multicenter, phase 3, randomized, non‐inferiority trial showed that 26 Gy in 5 fractions (over 1 week) was non‐inferior than 40 Gy in 15 fractions (over 3 weeks), and the normal tissue response assessed jointly by clinicians, patients, and photographs was safe.^19^ Yom et al. enrolled 306 cases of oropharyngeal cancer patients with p16‐positive and randomly divided into cisplatin‐based concurrent chemoradiotherapy (60 Gy of IMRT over 6 weeks with concurrent weekly cisplatin) and radiotherapy alone (60 Gy IMRT over 5 weeks). For concurrent chemoradiotherapy patients, 2‐year PFS was 90.5%; for IMRT, 2‐year PFS was 87.6% (*p* = 0.23). Acute effect and grade 3–4 late effect in CRT and IMRT alone was 79.6%, 52.4% (*p* < 0.001), and 21.3%, 18.1% (*p* = 0.56), respectively.^20^

The explorations of de‐intensification in treatment of NPC involved narrowing the margin of primary tumor and cervical target,^21^, ^22^ de‐escalating radiotherapy dose,^23^, ^24^ optimizing chemotherapeutic agents and course of treatment as well as deleting concurrent chemotherapy.^25^, ^26^, ^27^, ^28^, ^29^ A non‐inferiority prospective study based on 332 low‐risk III‐IVa NPC patients was performed and showed no significant differences on PFS, OS and the cumulative incidences of locoregional relapse or distant metastasis between two cycles and three cycles of DDP‐based CCRT groups. Patients in 2 cycles of DDP‐based concurrent chemoradiotherapy developed significantly less grade 3–4 mucositis, hyponatremia, hearing impairment and cervical skin fibrosis.^26^ Another multicenter, randomized, phase 3, non‐inferiority clinical trial enrolled 341 low‐risk NPC patients (II/T3N0) and divided into IMRT‐alone and CCRT group. The 3‐year failure‐free survival was 90.5% and 91.9% for IMRT‐alone and CCRT group, respectively. Similarly, there was no significant difference existed in terms of OS, LPFS, and DMFS. A significantly lower incidence of adverse events especially grade 3 or 4 hematological toxicities (leukopenia, neutropenia) and nonhematological toxicities (nausea, vomiting, anorexia, weight loss, mucositis) were observed in the IMRT‐alone group (17% vs. 46%). Simultaneously, the IMRT‐alone group had significantly better social function scores during radiotherapy.^27^ A meta‐analysis based on eight articles extracted data from 2605 NPC who received induction chemotherapy plus IMRT (IC + RT) or induction chemotherapy plus concurrent chemoradiotherapy (IC + CCRT), respectively. No statistically significant benefit was observed in terms of overall survival, local recurrence‐free survival, and distant metastasis‐free survival with IC + CCRT group. The proportion of grade 3–4 hematological toxicity was higher in the IC + CCRT group.^29^ Therefore, de‐intensifying or omitting concurrent chemotherapy in specified NPC patients seemed feasible. In this study, the omission of concurrent chemotherapy achieved satisfactory survival for T4 patients with 5‐year OS rates of 73.3%.

Dose on target volume was associated with local control significantly. Ng et al. analyzed 193 primary diagnosed non‐metastasis NPC patients, of which 93% had Stage III/IV disease. After median follow‐up 30 months, they proposed that at least 66.5 Gy to target volumes can lessen locoregional failure significantly.^30^ The irradiation dose of nasopharyngeal primary tumors is usually limited due to its special anatomical location. T1‐3 NPC always have better local tumor control since the target volume can get enough radiation dose without increasing the central nervous system (CNS) exposure. Inversely, advanced T4 is characterized by intracranial extension and/or involvement of CNS. The local failure‐free survival of T4 disease was significantly lower when compared with T1, T2, and T3 disease, while the incidence of CNS damage was higher than T1‐2 disease.^3^, ^4^, ^31^, ^32^, ^33^ Forty‐one cases of T4 NPC with intracranial invasion were analyzed by Xue. In order to avoid overdose irradiated of CNS, the maximum point dose (*D*_max_) for neurological organs was 55.9 (53.3–58.2) Gy, 43.9 (41.0–45.7) Gy, 56.4 (43.1–59.6) Gy, 54.6 (42.4–60.1) Gy, 54.5 (42.4–58.9) Gy, 65.1 (60.4–68.4), 64.7 (58.8–67.6) for brainstem, spinal cord, optic chiasm, affected side of optic nerve, unaffected side of optic nerve, affected side of temporal lobe and unaffected side of temporal lobe, respectively. While the minimum point dose for primary gross target volume was 55.2 (48.3–67.3) Gy, dose to 95% of the target volume was 61.6 (52.6–69.0) Gy.^32^ A total of 3328 NPC patients with stage III/IV performed IMRT in Hong Kong public oncology centers, of which 13.2% did not receive chemotherapy, most commonly due to advanced age, poor performance status, and patient's refusal. In phases III and IV, 83.3% and 87.2% received concurrent chemotherapy plus induction/adjuvant chemotherapy, respectively. The 5‐year, 8‐year LPFS rate and OS for T4 were 76.0%, 71.6% and 63.5%, 51.2% respectively.^3^ Wu et al. analyzed 614 NPC underwent IMRT and revealed that use of concurrent chemoradiotherapy plus induction/adjuvant chemotherapy was favorable for stage III/IV NPC patients. The 5‐year, 10‐year LPFS rate and OS for T4 NPC were 83.0%, 79.3%, and 68.1%, 59.2%, respectively.^4^ In our retrospective investigation, the 5‐year and 10‐year OS and LPFS rates for T4 NPC who did not receive concurrent chemotherapy were 73.8% and 59.3%, 87.5% and 79.3%, respectively. Similar conclusions have been obtained on other literatures that IMRT without concurrent chemotherapy in local advanced stage. A comparison of 5‐year LRFS and OS in T4 NPC among previous studies and our study have been listed in Table 4.

**TABLE 4:** The 5‐year LRFS and OS in T4 NPC among various studies.

Author	No. of T4 cases	LRFS (%)	OS (%)
Zhao et al.^34^	135	85.8	65.8
Ou et al.^2^	119	83.2	76.5
Wu et al.^4^	163	83.0	68.1
Huang et al.^5^	157	82.8	68.2
Chen et al.^35^	175	82.5	63.8
Au et al.^3^	612	76.0	63.5
Our study	145	86.1	73.7

Patients with N3 NPC are at high risk of distant metastasis and poor survival rate. However, few studies dedicated to analyze the effect of N‐classification among the T4 subgroup. Lin et al. investigated the survival impacts of various T‐classification on N3 stage and revealed that patients with T4 have worse OS (5‐year rates, 42.2% vs. 82.8%, *p* < 0.0001) and DMFS (5‐year rates, 57.2% vs. 77.7%, *p* = 0.0163) while compared with T1‐3N3 disease.^36^ Tsai et al. conducted a study on adjuvant chemotherapy after definitive concurrent chemoradiotherapy for N3 nasopharyngeal cancer. After classified the whole group of patients by T stage, they found that the 5‐year OS rate for T4N3 patients was 53.3%.^37^ Our study reported a 5‐year OS rate of 52.6% on the basis of deleting concurrent chemotherapy, which is close to the above two studies. Besides, Lee et al. proposed that the impact of concurrent chemotherapy on improving distant metastasis is insufficient.^38^ Patients with T4N3 are supposed to incorporate novel agents, such as targeted and immune oncology therapies to improve the treatment outcome. Multiple clinical studies are recruiting, such as Sichuan Cancer Hospital and Institute is performing a prospective, randomized, double‐blind, placebo‐controlled, multicenter phase II clinical study on the efficacy and safety of combined Nituzumab and IC for locally advanced NPC. Sun Yat‐sen University Cancer Center is carrying out a randomized, open‐label, phase II study to evaluate the efficacy and safety of Sintilimab plus capecitabine versus capecitabine alone as adjuvant therapy for patients with locoregionally advanced NPC.

Due to the deletion of concurrent chemotherapy during IMRT, decreasing side effects including mucosal reactions, nausea, and vomiting were observed.^28^, ^29^, ^39^ Wang et al. conducted a retrospective analysis of 332 patients with locally advanced NPC treated with induction chemotherapy and IMRT. According to the 2010 American Joint Committee for Cancer (AJCC 2010), patients classified as stage III, IVa, and IVb were 197 cases (59.3%), 86 cases (25.9%), and 49 cases (14.8%), respectively. For chemotherapy scheme, 39 cases (11.7%) of TPF, 128 cases (38.6%) of TP, 19 cases (5.7%) of GP, 10 cases (3.0%) of FP, and 16 cases (4.8%) of other. 123 cases (37.0%), 126 cases (38.0%), and 83 cases (25.0%) completed one, two, and three/four cycles of induction chemotherapy, respectively. After median follow‐up time of 65 months, 5‐year estimated rates of OS, locoregional relapse‐free survival, and DMFS were 82.5%, 93.4%, and 91.7%, respectively. During IMRT, grades 1, 2, 3, and 4 of mucositis or digestive tract reaction were 53.0%, 31.6%, 4.8%, 0% and 7.5%, 3.6%, 2.4%, 0%, respectively.^28^ Wang et al. followed up and analyzed 243 NPC patients who were sequentially treated with IC followed by IMRT and AC, of which 14 cases developed nasopharyngeal recurrence, 2 developed cervical region recurrence, 39 developed distant metastases. The 5‐year OS rate of stage III and IVa (AJCC 8th) was 86.5% and 56.5%.^39^

In summary, retrospective study showed the combination of IMRT with sequential chemotherapy deleting concurrent chemotherapy is an effective method. Prospective studies in low‐risk III‐IVA and II/T3N0 NPC did not observe significant lower efficacy by deleting concurrent chemotherapy. Our results showed that the combination of IMRT with sequential chemotherapy deleting concurrent chemotherapy in T4 NPC could be regarded as an effective method. Detects in our study were a single center and retrospective study. Future prospective and multicenter studies should be conducted to explore whether deletion of concurrent chemotherapy is possible for non‐low‐risk advanced nasopharyngeal carcinoma.

## AUTHOR CONTRIBUTIONS

**Yuming Zheng:** Data curation (equal); formal analysis (equal); software (equal); writing – original draft (equal). **Fen Xue:** Data curation (equal); formal analysis (equal); writing – original draft (equal). **Dan Ou:** Methodology (equal); software (equal); validation (equal). **Xiaoshuang Niu:** Investigation (equal); methodology (equal); validation (equal). **chaosu Hu:** Conceptualization (equal); funding acquisition (equal); project administration (equal); supervision (equal); writing – review and editing (equal). **Xiayun He:** Conceptualization (lead); funding acquisition (lead); project administration (lead); resources (lead); supervision (lead); validation (lead); writing – review and editing (lead).

## FUNDING INFORMATION

This work was supported by Shanghai Sailing Program (grant no: 21YF1408400), Scientific and Innovative Action Plan of Shanghai (grant no: 21Y11911900), and Key Clinical Specialty Project of Shanghai.

## CONFLICT OF INTEREST STATEMENT

The authors declare that they have no competing interests.

## Data Availability

N/A

## References

[1] GLOBAL . Cancer Observatory. 2020; Available from: https://gco.iarc.fr/

[2] Ou X , Zhou X , Shi Q , et al. Treatment outcomes and late toxicities of 869 patients with nasopharyngeal carcinoma treated with definitive intensity modulated radiation therapy: new insight into the value of total dose of cisplatin and radiation boost. Oncotarget. 2015;6(35):38381‐38397. doi:10.18632/oncotarget.5420 26485757 PMC4742007

[3] Au KH , Ngan RKC , Ng AWY , et al. Treatment outcomes of nasopharyngeal carcinoma in modern era after intensity modulated radiotherapy (IMRT) in Hong Kong: a report of 3328 patients (HKNPCSG 1301 study). Oral Oncol. 2018;77:16‐21. doi:10.1016/j.oraloncology.2017.12.004 29362121

[4] Wu LR , Liu YT , Jiang N , et al. Ten‐year survival outcomes for patients with nasopharyngeal carcinoma receiving intensity‐modulated radiotherapy: an analysis of 614 patients from a single center. Oral Oncol. 2017;69:26‐32. doi:10.1016/j.oraloncology.2017.03.015 28559017

[5] Huang J , Yang ZY , Wu B , et al. Long‐term therapeutic outcome and prognostic factors of patients with nasopharyngeal carcinoma receiving intensity‐modulated radiotherapy: an analysis of 608 patients from low‐endemic regions of China. Curr Med Sci. 2021;41(4):737‐745. doi:10.1007/s11596-021-2405-3 34403099

[6] He T , Yan RN , Chen HY , et al. Comparing the 7th and 8th editions of UICC/AJCC staging system for nasopharyngeal carcinoma in the IMRT era. BMC Cancer. 2021;21(1):327. doi:10.1186/s12885-021-08036-8 33785010 PMC8011200

[7] Chow JCH , Lee A , Bao KKH , et al. Cranial neuropathies in advanced nasopharyngeal carcinoma: neurological recovery after modern radiotherapy and systemic chemotherapy. Radiother Oncol. 2021;163:221‐228. doi:10.1016/j.radonc.2021.08.022 34506830

[8] Lee AWM , Tung SY , Ng WT , et al. A multicenter, phase 3, randomized trial of concurrent chemoradiotherapy plus adjuvant chemotherapy versus radiotherapy alone in patients with regionally advanced nasopharyngeal carcinoma: 10‐year outcomes for efficacy and toxicity. Cancer. 2017;123(21):4147‐4157. doi:10.1002/cncr.30850 28662313

[9] Wei Z , Zhang Z , Luo J , Li N , Peng X . Induction chemotherapy plus IMRT alone versus induction chemotherapy plus IMRT‐based concurrent chemoradiotherapy in locoregionally advanced nasopharyngeal carcinoma: a retrospective cohort study. J Cancer Res Clin Oncol. 2019;145(7):1857‐1864. doi:10.1007/s00432-019-02925-z 31062162 PMC11810258

[10] Qiu WZ , Huang PY , Shi JL , Xia HQ , Zhao C , Cao KJ . Neoadjuvant chemotherapy plus intensity‐modulated radiotherapy versus concurrent chemoradiotherapy plus adjuvant chemotherapy for the treatment of locoregionally advanced nasopharyngeal carcinoma: a retrospective controlled study. Chin J Cancer. 2016;35:2. doi:10.1186/s40880-015-0076-9 26739148 PMC4704429

[11] Chang H , Peng L , Tao YL , et al. Necessity of concurrent chemotherapy in N2‐3 nasopharyngeal carcinoma treated with neoadjuvant chemotherapy of ≥3 cycles followed by intensity‐modulated radiotherapy. Cancer Med. 2019;8(6):2823‐2831. doi:10.1002/cam4.2179 31006996 PMC6558596

[12] Liu Y , Yang L , Zhang S , Lin B . The efficacy and safety of concurrent chemoradiotherapy with induction chemotherapy vs. concurrent chemoradiotherapy alone for locally advanced nasopharyngeal carcinoma: a systematic‐review and meta‐analysis. Transl Cancer Res. 2022;11(5):1207‐1218. doi:10.21037/tcr-22-604 35706809 PMC9189232

[13] Zhang Y , Chen L , Hu GQ , et al. Final overall survival analysis of gemcitabine and cisplatin induction chemotherapy in nasopharyngeal carcinoma: a multicenter, randomized phase III trial. J Clin Oncol. 2022;40(22):2420‐2425. doi:10.1200/jco.22.00327 35709465

[14] Oliva M , Huang SH , Taylor R , et al. Impact of cumulative cisplatin dose and adjuvant chemotherapy in locally‐advanced nasopharyngeal carcinoma treated with definitive chemoradiotherapy. Oral Oncol. 2020;105:104666. doi:10.1016/j.oraloncology.2020.104666 32272384

[15] Xu T , Shen C , Ou X , He X , Ying H , Hu C . The role of adjuvant chemotherapy in nasopharyngeal carcinoma with bulky neck lymph nodes in the era of IMRT. Oncotarget. 2016;7(15):21013‐21022. doi:10.18632/oncotarget.7849 26942700 PMC4991508

[16] Kong FF , Ni MS , Zhai RP , Ying HM , Hu CS . Local control and failure patterns after intensity modulated radiotherapy with reduced target volume delineation after induction chemotherapy for patients with T4 nasopharyngeal carcinoma. Transl Oncol. 2022;16:101324. doi:10.1016/j.tranon.2021.101324 34953342 PMC8715109

[17] Trapani D , Franzoi MA , Burstein HJ , et al. Risk‐adapted modulation through de‐intensification of cancer treatments: an ESMO classification. Ann Oncol. 2022;33(7):702‐712. doi:10.1016/j.annonc.2022.03.273 35550723

[18] André T , Meyerhardt J , Iveson T , et al. Effect of duration of adjuvant chemotherapy for patients with stage III colon cancer (IDEA collaboration): final results from a prospective, pooled analysis of six randomised, phase 3 trials. Lancet Oncol. 2020;21(12):1620‐1629. doi:10.1016/s1470-2045(20)30527-1 33271092 PMC7786835

[19] Murray Brunt A , Haviland JS , Wheatley DA , et al. Hypofractionated breast radiotherapy for 1 week versus 3 weeks (FAST‐Forward): 5‐year efficacy and late normal tissue effects results from a multicentre, non‐inferiority, randomised, phase 3 trial. Lancet. 2020;395(10237):1613‐1626. doi:10.1016/s0140-6736(20)30932-6 32580883 PMC7262592

[20] Yom SS , Torres‐Saavedra P , Caudell JJ , et al. Reduced‐dose radiation therapy for HPV‐associated oropharyngeal carcinoma (NRG oncology HN002). J Clin Oncol. 2021;39(9):956‐965. doi:10.1200/jco.20.03128 33507809 PMC8078254

[21] Guo Q , Zheng Y , Lin J , et al. Modified reduced‐volume intensity‐modulated radiation therapy in non‐metastatic nasopharyngeal carcinoma: a prospective observation series. Radiother Oncol. 2021;156:251‐257. doi:10.1016/j.radonc.2020.12.035 33418007

[22] Tang LL , Huang CL , Zhang N , et al. Elective upper‐neck versus whole‐neck irradiation of the uninvolved neck in patients with nasopharyngeal carcinoma: an open‐label, non‐inferiority, multicentre, randomised phase 3 trial. Lancet Oncol. 2022;23(4):479‐490. doi:10.1016/s1470-2045(22)00058-4 35240053

[23] Xue F , Ou D , Ou X , Zhou X , Hu C , He X . Long‐term results of the phase II dose and volume de‐escalation trial for locoregionally advanced nasopharyngeal carcinoma. Oral Oncol. 2022;134:106139. doi:10.1016/j.oraloncology.2022.106139 36179488

[24] Mai HQ , Yang JH , Guo SS , et al. Reduced‐dose radiotherapy for pretreatment EBV DNA selected low‐risk stage III nasopharyngeal carcinoma: a single‐arm, phase II trial. J Clin Oncol. 2022;40(16):1.34793249

[25] Wang Y , Wang C , He S , et al. Induction chemotherapy regimen of docetaxel plus cisplatin versus docetaxel, cisplatin plus fluorouracil followed by concurrent chemoradiotherapy in locoregionally advanced nasopharyngeal carcinoma: preliminary results of an open‐label, noninferiority, multicentre, randomised, controlled phase 3 trial. EClinicalMedicine. 2022;53:101625. doi:10.1016/j.eclinm.2022.101625 36060517 PMC9433608

[26] Li XY , Luo DH , Guo L , et al. Deintensified chemoradiotherapy for pretreatment Epstein‐Barr virus DNA‐selected low‐risk locoregionally advanced nasopharyngeal carcinoma: a phase II randomized noninferiority trial. J Clin Oncol. 2022;40(11):1163‐1173. doi:10.1200/jco.21.01467 34990291

[27] Tang LL , Guo R , Zhang N , et al. Effect of radiotherapy alone vs radiotherapy with concurrent chemoradiotherapy on survival without disease relapse in patients with low‐risk nasopharyngeal carcinoma: a randomized clinical trial. Jama. 2022;328(8):728‐736. doi:10.1001/jama.2022.13997 35997729 PMC9399866

[28] Fangzheng W , Chuner J , Haiyan Q , et al. Survival without concurrent chemotherapy for locoregionally advanced nasopharyngeal carcinoma treated with induction chemotherapy plus intensity‐modulated radiotherapy: single‐center experience from an endemic area. Medicine (Baltimore). 2019;98(51):e18484. doi:10.1097/md.0000000000018484 31861031 PMC6940191

[29] Wang Q , Xu G , Xia Y , et al. Comparison of induction chemotherapy plus concurrent chemoradiotherapy and induction chemotherapy plus radiotherapy in locally advanced nasopharyngeal carcinoma. Oral Oncol. 2020;111:104925. doi:10.1016/j.oraloncology.2020.104925 32721816

[30] Ng WT , Lee MC , Hung WM , et al. Clinical outcomes and patterns of failure after intensity‐modulated radiotherapy for nasopharyngeal carcinoma. Int J Radiat Oncol Biol Phys. 2011;79(2):420‐428. doi:10.1016/j.ijrobp.2009.11.024 20452132

[31] Cao CN , Luo JW , Gao L , et al. Clinical outcomes and patterns of failure after intensity‐modulated radiotherapy for T4 nasopharyngeal carcinoma. Oral Oncol. 2013;49(2):175‐181. doi:10.1016/j.oraloncology.2012.08.013 23021729

[32] Xue F , Hu CS , He XY . Effects of dosimetric inadequacy on local control and toxicities in the patients with T4 nasopharyngeal carcinoma extending into the intracranial space and treated with intensity‐modulated radiotherapy plus chemotherapy. Chin J Cancer. 2017;36(1):76. doi:10.1186/s40880-017-0245-0 28931426 PMC5607564

[33] Ng WT , Lee MC , Chang AT , et al. The impact of dosimetric inadequacy on treatment outcome of nasopharyngeal carcinoma with IMRT. Oral Oncol. 2014;50(5):506‐512. doi:10.1016/j.oraloncology.2014.01.017 24529762

[34] Zhao W , Lei H , Zhu X , Li L , Qu S , Liang X . Investigation of long‐term survival outcomes and failure patterns of patients with nasopharyngeal carcinoma receiving intensity‐modulated radiotherapy: a retrospective analysis. Oncotarget. 2016;7(52):86914‐86925. doi:10.18632/oncotarget.13564 27894100 PMC5349963

[35] Chen Y , Zhang Q , Lu T , et al. Prioritizing sufficient dose to gross tumor volume over normal tissue sparing in intensity‐modulated radiotherapy treatment of T4 nasopharyngeal carcinoma. Head Neck. 2023;45(5):1130‐1140. doi:10.1002/hed.27315 36856128

[36] Lin TY , Lan MY , Tsou HH , et al. Survival impacts of different nodal characteristics and T‐classification in N3 nasopharyngeal carcinoma patients. Oral Oncol. 2020;108:104820. doi:10.1016/j.oraloncology.2020.104820 32531741

[37] Tsai MH , Wu SY , Lu HH , Yu T , Tsai ST , Wu YH . Improved overall survival is associated with adjuvant chemotherapy after definitive concurrent chemoradiotherapy for N3 nasopharyngeal cancer. Sci Rep. 2022;12(1):13390. doi:10.1038/s41598-022-16422-w 35927415 PMC9352661

[38] Lee AW , Tung SY , Ngan RK , et al. Factors contributing to the efficacy of concurrent‐adjuvant chemotherapy for locoregionally advanced nasopharyngeal carcinoma: combined analyses of NPC‐9901 and NPC‐9902 trials. Eur J Cancer. 2011;47(5):656‐666. doi:10.1016/j.ejca.2010.10.026 21112774

[39] Wang P , Dong F , Cai C , Ke C . Treatment outcomes of induction chemotherapy combined with intensity‐modulated radiotherapy and adjuvant chemotherapy for locoregionally advanced nasopharyngeal carcinoma in Southeast China. Medicine (Baltimore). 2021;100(33):e27023. doi:10.1097/md.0000000000027023 34414997 PMC8376380

